# Smartwatch based automatic detection of out-of-hospital cardiac arrest: Study rationale and protocol of the *HEART-SAFE* project

**DOI:** 10.1016/j.resplu.2022.100324

**Published:** 2022-11-10

**Authors:** Patrick Schober, Wisse M.F. van den Beuken, Beat Nideröst, Tom A. Kooy, Steve Thijssen, Carolien S.E. Bulte, Bregje A.A. Huisman, Pieter R. Tuinman, Alexander Nap, Hanno L. Tan, Stephan A. Loer, Gaby Franschman, Roelof G. Lettinga, Derya Demirtas, Susanne Eberl, Hans van Schuppen, Lothar A. Schwarte

**Affiliations:** a Amsterdam UMC location Vrije Universiteit Amsterdam, Department of Anesthesiology, Amsterdam, the Netherlands; b Helicopter Emergency Medical Service Lifeliner 1, Amsterdam, the Netherlands; c 111 b.v., Elburg, the Netherlands; d Stan b.v., Elburg, the Netherlands; e Wavy Health b.v., Groningen, the Netherlands; f Hospice Kuria, Amsterdam, the Netherlands; g Amsterdam UMC location Vrije Universiteit Amsterdam, Department of Intensive Care Medicine, Amsterdam, the Netherlands; h Amsterdam UMC location Vrije Universiteit Amsterdam, Department of Cardiology, Amsterdam, the Netherlands; i Amsterdam UMC location University of Amsterdam, Department of Cardiology, Amsterdam, the Netherlands; j Regional Ambulance Service Kennemerland, Haarlem, the Netherlands; k University Medical Centre Groningen, Department of Anesthesiology Groningen, the Netherlands; l UMCG Ambulance Care, Tynaarlo, the Netherlands; m University of Twente, Department of Industrial Engineering and Business Information Systems, Enschede, the Netherlands; n Amsterdam UMC location University of Amsterdam, Department of Anesthesiology, Amsterdam, the Netherlands

**Keywords:** Out-of-Hospital Cardiac Arrest, Photoplethysmography, Smartwatch

## Abstract

Out-of-hospital cardiac arrest (OHCA) is a leading cause of mortality. Immediate detection and treatment are of paramount importance for survival and good quality of life. The first link in the ‘chain of survival’ after OHCA – the early recognition and alerting of emergency medical services – is at the same time the weakest link as it entirely depends on witnesses. About one half of OHCA cases are unwitnessed, and victims of unwitnessed OHCA have virtually no chance of survival with good neurologic outcome. Also in case of a witnessed cardiac arrest, alerting of emergency medical services is often delayed for several minutes. Therefore, a technological solution to automatically detect cardiac arrests and to instantly trigger an emergency response has the potential to save thousands of lives per year and to greatly improve neurologic recovery and quality of life in survivors.

The HEART-SAFE consortium, consisting of two academic centres and three companies in the Netherlands, collaborates to develop and implement a technical solution to reliably detect OHCA based on sensor signals derived from commercially available smartwatches using artificial intelligence. In this manuscript, we describe the rationale, the envisioned solution, as well as a protocol outline of the work packages involved in the development of the technology.

## Introduction

Out-of-hospital cardiac arrest (OHCA) is a leading cause of mortality the United States and Europe,^1^, ^2^ accounting for 15–20 % of deaths.^3^ The first link in the so-called ‘chain of survival’ after OHCA is the early recognition of cardiac arrest and alerting of emergency medical services (EMS). This first link is at the same time the weakest link as it entirely depends on witnesses who quickly recognize OHCA and promptly alert the EMS. However, about half of OHCA cases is unwitnessed, and these victims currently have virtually no chance of survival with good neurologic outcome.^4^ Even among witnessed cases, alerting of EMS by bystanders is frequently delayed until several minutes after a cardiac arrest.^5^ The odds of survival and good neurologic outcome dramatically decline with each minute of delay until initiation of cardiopulmonary resuscitation (CPR),^6^, ^7^, ^8^ such that immediate detection and early treatment of cardiac arrest are of paramount importance for survival and good quality of life. A technological solution to automatically detect cardiac arrests and to instantly trigger an emergency response thus has the potential to save thousands of lives per year and to greatly improve neurologic recovery and quality of life in survivors.

The HEART-SAFE (**H**ome **E**mergency **A**lerting and **R**esponse **T**echnology – **S**urvive **A F**atal **E**vent) consortium has initiated a project in the Netherlands, in which two academic centres (a University Hospital and a Technical University) as well as three companies (with expertise in sensor technology, artificial intelligence and alerting of citizen responders), collaborate to develop and implement a technical solution to reliably detect OHCA based on sensor signals derived from commercially available smartwatches, and to automatically alert EMS dispatch centres and citizen CPR volunteers (https://HartslagNu.nl). The project makes use of a data-driven machine learning approach to identify patterns related to cardiac arrest in signal data from smartwatch sensors, such as absence of pulsatile blood flow, fall detection and absence of movement. We envision that in no more than 5 years, individuals who wish to protect themselves from deadly delays in recognizing a cardiac arrest will be increasingly able to do so in a reliable, affordable, and simple manner.

To achieve this, we release an open-access platform that connects all components of the chain of survival, from monitoring of individuals for OHCA in their own living environment to advanced life support offered by EMS if required. The purpose of this manuscript is to provide a description of the rationale and a protocol outline of the steps that we are taking to develop this technology, which has the potential to revolutionize the way that EMS and citizen CPR volunteers will be alerted in cases of OHCA in the (near) future.

## Methods

### Objective and general overview

**Fig. 1:**
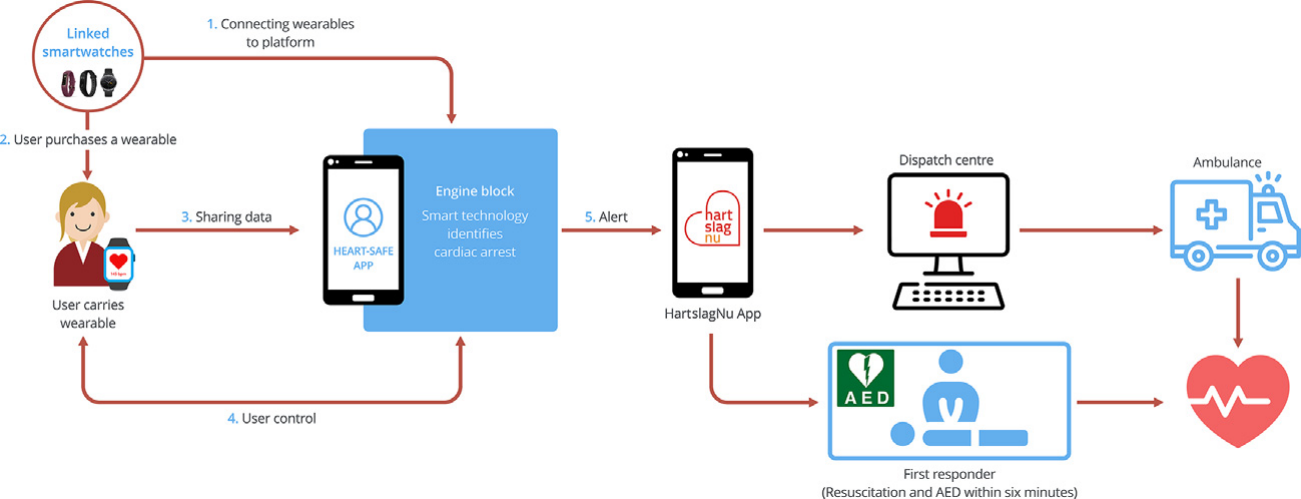
Schematic overview over the technology. The open access data platform will allow connection of a variety of smartwatches (and will be available for other wearables capable of detecting OHCA should such devices be developed by other research groups in the future), and the user of a connected smartwatch will be continuously monitored for OHCA. In the case that OHCA is detected, citizen rescuers (in the Netherlands: HartslagNu network) as well as Emergency Medical Services will be activated, and geolocation data is automatically transmitted.

The main objective is to develop and implement a technical solution to automatically and reliably detect OHCA, and to trigger an immediate emergency response as schematically depicted in Fig. 1. We expect this technology to markedly decrease the time to CPR, both in patients with unwitnessed but also in patients with witnessed cardiac arrest, potentially saving many thousands of lives each year and improving neurologic outcomes. This solution comprises two main components:

Component 1: Using artificial intelligence (AI), we develop an *open-source cardiac arrest detection algorithm* based on signals derived from photoplethysmography (PPG) and additional sensors in commercial smartwatches as described in more detail below.

Component 2: We deliver a *device-independent interface (the “engine block”)* between component 1 and the Dutch citizen-rescuer system HartslagNu as well as EMS dispatch centres. As part of the engine block, we develop the HEART-SAFE app, which provides audio-visual alerting of users and bystanders, and a stop button for cancelling a false alarm. The engine block will also provide GDPR (General Data Protection Regulation of the European Union) compliant profile storage, providing essential medical data to rescuers and additional information needed to access the patient, such as geolocation data.

**Fig. 2:**
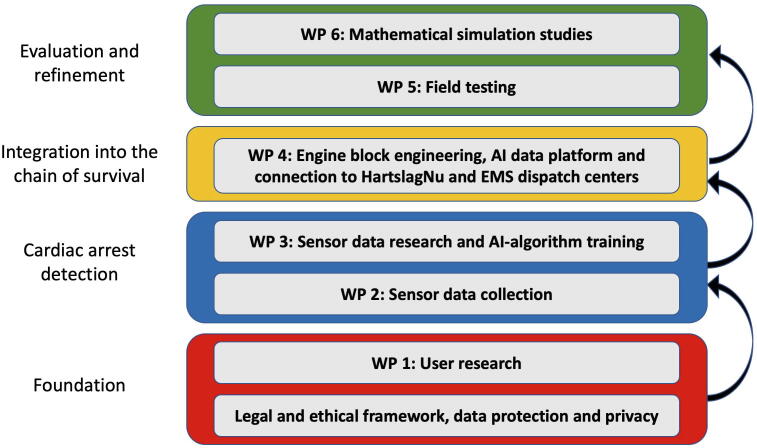
Schematic outline of the stepwise approach and work packages (WP) involved in the development of the technology. The legal and ethical framework as well as user research (WP1) form the foundation that is required to proceed to the development of the cardiac arrest detection algorithm. This development relies on the collection of sensor data (WP2) and on signal research as well as training of the artificial intelligence (AI) based algorithm to detect cardiac arrest (WP3). Subsequently, the algorithm will be integrated into the chain of survival, connecting the user via an app and a data platform to the HartslagNu citizen rescuer alerting system as well as EMS dispatch centres (WP4). The technology is now ready for evaluation and further refinements in field testing (WP5) and mathematical simulation studies (WP6).

The technical developments will be accompanied by extensive research. User research, signal-data research, a field lab and simulation studies will be performed to understand user needs, to train the AI-algorithm, to evaluate the technology, and to estimate the impact on survival and quality of life, as further described below and as illustrated in Fig. 2.

### Work packages, methods and study protocol outline

Work package 1: User research. In order to develop a technology and service that is well aligned with the needs of prospective users and taking into account user diversity, we initially perform user research in those users who would potentially use a smartwatch to protect themselves from delays in detection of OHCA (primary users), as well as those who will be alerted in case of a detected OHCA, including citizen rescuers and ambulance services (secondary users). We are particularly interested in the features that the technology should have from the users’ perspective as well as in the motives of users who would not want to use the technology, such that we can detect and address obstacles early in the development process. To achieve this, different potential primary and secondary user groups, including current smartwatch users, patients at increased risk of cardiac arrest, citizen CPR volunteers, ambulance personnel and EMS dispatchers will be surveyed.

Work package 2: Sensor data collection. In order to gain a fundamental understanding of the various sensor data characteristics (e.g., signal strength, artifacts under various conditions, biological relevance, etc) and to subsequently train the algorithm to detect cardiac arrest, a rich set of sensor data collected under varying conditions in individuals with and without cardiac arrest is necessary. In contrast to hypothesis-driven clinical studies, in which the required sample size to detect a certain effect size at a certain alpha level with certain power can be calculated *a priori*,^9^ such calculations are not straightforward for machine learning approaches.^10^ The amount of data depends on a variety of unknown factors, including the signal-to-noise ratio, the relative contribution of different sensors and complexity of the classification model, and the incidence of cardiac arrests in our patient population. Based on a previous study in which a machine learning algorithm was trained to detect agonal breathing,^11^ and considering a trade-off between the ‘more-is-better’ paradigm in machine learning on the one hand and feasibility of data collection on the other hand, we target at a sample size of about 400 datasets.

Smartwatch sensor data will be collected in a diverse study population of healthy individuals as well as patients, including individuals in whom (A) a circulatory arrest in the arm can be simulated by temporarily restricting blood flow with a tourniquet, (B) in whom a temporary cardiac arrest is artificially induced during routine patient care as detailed below, (C) patients who present with cardiac arrest and (D) patients at high risk of experiencing a cardiac arrest. Specifically, we obtain smartwatch sensor data in the following patient or participant categories:

A. In healthy individuals, an inflatable tourniquet is used to temporarily restrict blood flow to the arm, thus mimicking a crucial aspect of circulatory arrest.
B. In patients undergoing cardiac surgery with cardiopulmonary bypass or undergoing certain interventions such as testing of an implantable cardioverter-defibrillator, a temporary cardiac arrest is artificially induced as part of the clinical treatment. This provides the opportunity to obtain cardiac arrest data and observe how the signal changes at onset of cardiac arrest.
C. Patients with cardiac arrest presenting to the emergency department allow training the algorithm to distinguish absence of pulsations before return of spontaneous circulation (ROSC) from presence of pulsations after ROSC.
D. Patients at high risk of cardiac arrest include patients at the intensive and cardiac care unit, the catheterization laboratory, patients with previous recurrent episodes of ventricular fibrillation after ICD implantation, as well as patients with palliative care at a hospice.

Work package 3: Cardiac arrest detection algorithm. Using the sensor data collected in work package 2, we will train and validate the algorithm to detect cardiac arrest. More specifically, this work includes:

• Fundamental study of baseline signal characteristics and impact of artifacts on readings.
• Basic proof of principle on test cases under various conditions and using various sensors.
• Identifying patterns in the raw sensor data that can be used to distinguish spontaneous circulation (e.g., sensor signals related to pulsatile blood flow or active movement, see below) as well as deliberate loss of normal pulsatile blood flow signal events (e.g., when a user removes the sensor from his body) on one side, from cardiac arrest events on the other side.
• Selecting the most appropriate signal processing components and pattern recognition algorithm and setting basic parameters.
• Tuning the signal processing components and training the pattern recognition algorithm using the collected and labelled sensor data, minimizing false positives and false negatives.
• Using cross-validation techniques to assess discriminative ability of the algorithm in validation datasets and to tune hyperparameters.

The result of this iterative process is the final algorithm which can be subsequently refined and evaluated for diagnostic accuracy in ‘real-life’ data in work package 5 as described below.

While we will explore all available sensor signals for their ability to detect (absence of) spontaneous circulation, the algorithm is primarily based on PPG data. PPG is an established medical technology, ubiquitously used in intensive and acute care, and nowadays also routinely integrated into smartwatches. PPG is capable of continuously monitoring the pulse rate, oxygen saturation (pulse oximetry) and additional physiologic parameters by measuring changes in light absorption at characteristic wavelengths during pulsatile blood flow generated by the heartbeat.^12^, ^13^ Any circulatory arrest – regardless of aetiology or demographic characteristics of the patient – is characterized by absence of pulsatile blood flow, which can reliably be detected by PPG.^14^, ^15^ Additional sensors are expected to further enhance the diagnostic accuracy and to avoid false positive alarms. For example, patients in cardiac arrest typically do not move at all, and may fall after onset of cardiac arrest if in a standing position, which can be detected with accelerometric and gyroscope sensor data.^16^ A portion of patients do still move due to agonal breathing after onset of cardiac arrest, which can be detected by characteristic movement patterns or acoustic signals via the integrated microphone.^11^ Therefore, rather than relying on a single sensor signal, we aim to identify characteristic signal patterns that are highly suggestive of cardiac arrest, such as a combination of absence of pulsations and either agonal breathing or absence of movement.

In this work package, we target at a high diagnostic accuracy with a sensitivity to detect cardiac arrests of at least 99 % and a specificity of at least 99.9 %. However, as the data obtained during field testing (work package 5) will allow further optimization of the diagnostic accuracy, a sensitivity of 90 % and a specificity of 95 % will suffice to proceed.

Work package 4: Engine block engineering. For the ‘engine block’ that will drive the entire system, one of the companies involved in this project will provide their dHealthAI data platform, which is a proven, production-ready service that offers GDPR compliant user information and signal storage, a high performant big data processing engine, and an AI platform that can be used for state-of-the-art signal analysis and pattern recognition applications. The final algorithm developed in work package 3 will be an integral part of this data platform.

To integrate the OHCA detection algorithm into the chain of survival, another company within our consortium is constructing an interface between the dHealthAI environment and the fully operational HartslagNu citizen rescuer alerting system as well as EMS dispatch centres. In case of a detected cardiac arrest, EMS as well as citizen rescuers are automatically alerted. In this work package, we will also develop the HEART-SAFE app as the interface between the user and the engine block, which allows for communication with smartwatches and provides a strong audible alarm in case of a cardiac arrest. The alarm alerts bystanders of a medical emergency and alerts the user who can cancel the alarm via a stop button in case of a false alarm. A final part of the engine block is GDPR compliant user profile storage. In the event of OHCA, these data provide HartslagNu and the EMS dispatch centres essential information about the patient, including the current location based on Global Positioning System (GPS) data. The user profile is extensible to store additional parameters, such as or the location of access codes for IoT smart door locks to allow access to the patient.

Work package 5: Field testing. In cooperation with our extensive network within the ambulance landscape in the Netherlands, we assess the diagnostic accuracy (sensitivity and specificity to detect cardiac arrest)^17^ and refine the technology in settings in which the technology is to be used after implementation, i.e., in non-hospitalized individuals in the home setting or when wearing the device outside home. This field test targeting at 10,000–25,000 smartwatch users at risk of cardiac arrest will not only focus on optimizing the reliability of the algorithm itself, but also on technical and non-technical aspects of using the technology in a ‘real-world’ setting. This includes an evaluation of the technical connections between the smartwatches, data-platform, HartslagNu and EMS dispatch centres, as well as an evaluation of Human Factors and Ergonomics (HFE) related to the use of the technology. All primary users as well as those secondary users responding to a detected cardiac arrest (citizen rescuers, EMS personnel) will be surveyed in order to identify remaining weak links within the chain of survival and to estimate the psychological impact and burden of using this technology.

Work package 6: Mathematical simulation studies will be used to (1) identify for which target groups the proposed technology is most effective, (2) fine-tune parameters of the alert strategy, and (3) estimate the impact on survival and quality of life after cardiac arrest. This simulation environment allows fine-tuning parameters and optimization of our solution. As a secondary aim, economic impacts can be estimated (i.e., Health Technology Assessment).

First, we build a computer simulation model to evaluate the expected health outcomes for a cohort of individuals living in selected pilot regions over a 10-year period and over a life-time horizon. Depending on the availability of patient-level data, this will either be a cohort model (i.e., Markov state-transition model) or a patient-level model (for example a microsimulation or discrete event simulation model). Model validation consists of technical model verification and face validity checks with experts. With this model, we compare lifetime health outcomes under the status-quo and under the early alert strategy for various patient groups under various parameters of the alerting strategy. Moreover, we estimate the decrease in time-to-CPR and time-to-defibrillation as a result of early detection of cardiac arrest. Using survival curves from literature and our resuscitation database as well as simulated CPR and defibrillation time, we can then estimate the proposed technology's impact on survival, and on quality of life following initial survival. Furthermore, it will provide an estimate of the incremental cost per additional cardiac arrest survived (>30 days, in good condition) as well as the incremental cost per quality adjusted life year (QALY) gained. The economic evaluation will not only consider costs and benefits of detecting cardiac arrests, but also the costs and other consequences (e.g., unavailability of ambulance crews for other emergencies) of false alarms.

### Ethical aspects, informed consent and consortium governance

As parts of the research do not fall under the *Dutch Medical Research Involving Human Subjects Act* (e.g., user research) whereas other parts do (e.g., field test of a medical device), several protocols have been and will be filed, respectively, with the Medical Research Ethics Committee (MREC) of the Amsterdam UMC. Ethical aspects of the project have been extensively discussed within the consortium, as well as with the ethicist of the MREC. Given the negligible risks and minimal burden involved for patients in the clinical studies on the one hand and the large potential benefit of the technology, we consider the risk–benefit ratio of this project as extremely favourable. A data management plan has been filed in cooperation with the Department Research Support / Research Data Management of Amsterdam UMC, and privacy aspects have been addressed in collaboration with the Data Protection Officer. Informed consent will be obtained from all participants in clinical studies or from their legal representative in case of mental incompetence (e.g., ICU patients). In the latter case, deferred patient consent will be sought as soon as practicable.

The consortium is governed by a steering group consisting of a representative from each consortium partner on the basis of the signed Consortium Agreement, with the primary aim to make strategic decisions, to provide scientific and technical oversight, and to coordinate the activities. Regular input from a user committee consisting of representatives of different user groups and other stakeholders will be sought.

## Discussion

OHCA affects more than 1,000 patients per day alone in the United States, and about one half of the cases are unwitnessed.^4^ A technical solution to automatically detect OHCA and to alert EMS will for the first time provide victims of unwitnessed OHCA a realistic chance of survival in good condition, and will likely also markedly decrease the time to treatment in cases of witnessed OHCA. A very recent systematic review described the diagnostic test performance of different sensor technologies that could potentially be used for the detection of cardiac arrest,^18^ but to the best of our knowledge, a technology that actually detects OHCA and alerts EMS has not been previously developed. Chan and colleagues have trained a support vector machine algorithm to reliably detect agonal breathing^11^; however, a large proportion of patients with OHCA do not have agonal breathing, and the study was not designed to include automatic alerting of EMS. Inspired by ideas of different consortium members and by a project call of the Dutch Heart Foundation, we therefore initiated a comprehensive project in which we train an algorithm to detect cardiac arrest primarily based on the detection of pulsatile blood flow generated by the heartbeat using PPG sensors in commercial smart watches. Absence of pulsatile blood flow is a key characteristic of all cardiac arrests, irrespective of the aetiology or patient characteristics. Moreover, we integrate the algorithm into the chain of survival, allowing for automatic alerting of EMS and citizen CPR rescuers. The technical developments are accompanied by extensive research activities, including user research, sensor data research, field testing as well simulation research. In a nutshell, this manuscript describes the rationale and presents an outline of the different work-packages involved. Future manuscripts will describe results of the clinical studies, field testing as well as simulation studies.

## Funding

The project is supported by a grant from the Top Consortia for Knowledge and Innovation’s (TKI) office of the 10.13039/100016036Dutch Life Sciences & Health (**LSH**) Top Sector (Health Holland), as well as in-kind contributions of all consortium partners.

## Author contributions

All authors participated in conceiving the study project. PS obtained research funding. LAS and PS drafted the manuscript, and all authors contributed substantially to its revision. PS takes responsibility for the paper as a whole.

## Declaration of Competing Interest

Beat Nideröst is CTO of 111b.v., Tom A. Kooy is International Business Developer at Stan b.v., Steve Thijssen is Co-Founder of Wavy Assistant b.v. All three companies are involved in the development of the proposed technology within the HEART-SAFE consortium. Hans van Schuppen reports grants to his institution from the Zoll Foundation and Stryker Emergency Care, both outside the submitted work.
